# 

**Published:** 1890-04

**Authors:** 


### CONTENTS:

ARTICLE I.—Ether, Chloroform or Nitrous Oxide. By George R. Fowler, M. D. - - - - - - 529

" II.—Professional Ethics and Honor. By W. Storer How, D.D.S. - - - - - - - - 540

" III.—Chloralamid. By Chas. H. Steele, A. M., M. D. - 549

" IV.—Care of the Deciduous Teeth. By F. S. Maxwell, D.D.S. - - - - - - - - 554

" V.—The Teeth and Orad Cavity of Pregnant Women. 559

" VI.—Cases of Epulis. By J. F. Colyer, M.R.C.S., L.D.S., Etc. - - - - - - - - 563

" VII.—Infection by Physicians. - - - - - 565

" VIII.—Tobacco-Smoking and Disease. - - - 566

Still Another New Hypnotic. - - - - - - 558

### EDITORIAL.

Baltimore College of Dental Surgery. - - - - - - 568

The Way to Educate Medical Students. - - - - 570

### MONTHLY SUMMARY.

Synthetic Carbolic Acid. - - - - - - - - 572

Eruption of Teeth in Old Age. - - - - - - 572

The Stomach-brush. - - - - - - - - 573

A Simple Regulating Appliance. - - - - - - 574

The Influence of Heredity in the Causation of Tuberculosis of Bone. - - - - - - - - - - 575

Goodness of Seeds. - - - - - - - 575

Thymol Dentifric. - - - - - - - - 576

A Popular Belief in the Contagiousness of Phthisis. - - 576

